# Supplementary material for: Critical Review of the Methodological Shortcoming of Ambulatory Blood Pressure Monitoring and Cognitive Function Studies
Source: Clocks Sleep. 2025 Mar 6;7(1):11. doi: 10.3390/clockssleep7010011 (PMC11941602; doi:10.3390/clockssleep7010011)
Supplement: Supplementary file 1 [file clockssleep-07-00011-s001.zip › clockssleep-3327322-supplementary.pdf]

Table S1. Characteristics of studies examining ABPM patterns and cognitive function.

First Author (Year)	Study Question	Study Type	Sample Size (% female)	Age (year)	Race	Cognitive Status at Recruitment	Cognitive Assessment[a]	Blood Pressure Status at Recruitment
Cani I (2022) [7]	Describe cognitive profile in patients with idiopathic autonomic failure	Cross-sectional	23 (30%)	Not specified	Not specified	Not in criteria	CI[b] was defined as an abnormal score on at least one test of the NPS[b] without specifying cognitive domains	Not in criteria
Chen HF (2013) [13]	Examine circadian rhythm of arterial BP[b] in AD[b] patients without hypertension	Cross-sectional	318 (46%)	76[c]	Not specified	AD[b] patients and healthy controls	NINCDS-ADRDA[b] criteria	Without hypertension[d]
Cicconetti P (2003) [14]	Investigate relationship between non-dipping BP[b] pattern and cognitive function in early hypertension	Cross-sectional	40 (65%)	62.9[c]	Not specified	No neurological diseases	MMSE[b] and ERPs[b] (N2, P300 latencies)	Newly diagnosed grade 1 and 2 hypertension[d]
Cicconetti P (2004) [15]	Investigate relationship between circadian BP[b] pattern and cognitive function in elderly with recently diagnosed hypertension	Cross-sectional	30 (90.0%)	68.3[c]	Not specified	No dementia	MMSE[b] and ERPs[b] (N2, P300 latencies)	Recently diagnosed grade 1 or 2 hypertension[d]
Daniela M (2023) [8]	Evaluate BP using 24h ABPM[b] in AD[b] and VaD[b] patients compared to healthy controls	Cross-sectional	90 (51.1%)	74.7	Not specified	30 AD[a], 30 VaD[a], 30 healthy controls	AD[b]: NINCDS-ADRDA[b] criteria; VaD: NINDS-AIREN[b] criteria, Hachinski score, CT[b]/MRI[b]	Not in criteria
Ghazi L (2020) [9]	Determine association between ABPM[b], cognitive function, physical function, and frailty in CKD[b] patients	Longitudinal (Cognitive follow-up after 4 years)	1,502 (44%)	63±10	45% non-Hispanic white, 39% non-Hispanic black, 12% Hispanic	Not in criteria	3MS[b]	Not in criteria
Gregory MA (2016) [16]	Determine if differences in cognitive and gait performance exist between older adults with normal vs. reduced BP[b] dipping status	Cross-sectional	115 (63%)	71.7±6.9	96% Caucasian	Without dementia	MoCA[b], MMSE[b], TMT[b], DSST[b], verbal fluency tasks, and AVLT[b]	> 180/100 mmHg or < 100/60 mmHg excluded
Guo H (2010) [17]	Investigate association of circadian BP[b] variation with MCI[b] in community-dwelling persons	Cross-sectional	144 (66%)	68 ± 7	Not specified	No definitive dementia	MCIS[b]	Without antihypertensive
Kececi Savan D (2016) [18]	Determine relationship between ABPM[b] and cognitive functions in elderly hypertensive patients	Cross-sectional	91 (77%)	71.9[c]	Not specified	Without antidemential medication	sMMT[b]<24=MCI/early dementia)	Hypertensive[d]
Kim JE (2009) [19]	Examine relationships between ABPM[b] patterns, subcortical ischemic lesions, and cognitive impairment	Cross-sectional	109 (42.2%)	69.9±4.12	Not specified	SvMCI[b], SVaD[b], or healthy controls	DSM[b]-IV, neuropsychological tests, CDR[b], I-ADL[b], Hachinski score, MRI[b] evidence of subcortical lesions	Some with hypertension[d]
Komori T (2016) [20]	Examine if abnormal circadian BP[b] rhythm is associated with MCI[b] in heart failure patients	Cross-sectional	444 (38.5%)	68±13	Not specified	Excluded those with documented dementia	MMSE[b]<26=MCI[b]	Not in criteria
Li XF (2017) [21]	Analyze correlation between cognitive impairment and ABPM[b] in patients with cerebral small vessel disease	Cross-sectional	108 (47.2%)	67.7[c]	Not specified	Healthy and cognitive impairment	MoCA[b]<23=CI[b]	refractory hypertensions were excluded
Mahmoud KS (2014) [22]	To test the correlation of ABPM[b] to cognitive function in elderly hypertensive patients	Cross-sectional	77 (46.8%)	69	Not specified	No neurological disorders	MMSE[b], MRI[b]	With history of hypertension and control group
Ohya Y (2001) [23]	Study the relationship among activity of daily living, cognitive function, and ABPM[b] in the elderly	Cross-sectional	99 (78%)	79.8±10.1	Not specified	AD[b] and neuronal degenerative disease were excluded	MMSE[b]	Without antihypertensive

Okuno J (2003) [24]	Investigate association between fall of nocturnal BP[b] and cognitive impairment in elderly subjects	Cross-sectional	204 (69.1%)	75.2±7.2	Not specified	People with severely impaired cognition were excluded	MMSE[b]≤23=CI[b]	Not in criteria
Paganini-Hill A (2019) [25]	Analyze relationship between BP[b] variables and cognition in 90+ year-olds	Cross-sectional	121 (63%)	93	All Caucasian except one Asian	Not in criteria	VFT[b] (Animal, Letter F), BNT[b], CVLT[b], TMT[b], Clock Drawing, CERAD[b] Construction, Digit Span, MMSE[b], 3MS[b], CDR[b], MRI[b]	Not in criteria
Shim YS (2022) [10]	Investigate ABPM[b] profiles and MRI[b] findings of cerebral small-vessel disease in older adults with cognitive complaints	Cross-sectional	174 (68.4%)	75.36±7.13	Not specified	SCD[b], MCI[b], or AD[b]	MMSE[b], CDR[b], CDR-SB[b], SNSB[b], MRI[b]	Not in criteria
Sierra C (2015) [26]	Investigate relationship between circadian BP[b] pattern and cognitive function in middle-aged essential hypertensive patients	Cross-sectional	56 (34%)	54.3±3.1	Not specified	Not in criteria	attention/working memory (Digit Span), logical/visual memory (WMS[b])	Never-treated essential hypertensive[d]
Suzuki R (2011) [27]	Investigate relationships between sleep disturbance, ADL[b], and ABPM[b] patterns in institutionalized dementia patients	Cross-sectional	107 (70.1%)	76.3±9.2	Not specified	Institutionalized dementia patients	DSM-III R, Hachinski Score, MMSE	Not in criteria
Tadic M (2019) [28]	Assess relationships between absolute and individual residual BP[b] variability and cognitive function in general population	Cross-sectional	471 (47%)	63±5.7	Not specified	Not in criteria	MMSE[b]	Not in criteria
Tan X (2021) [11]	Examine if nocturnal dipping pattern of systolic BP[b] was associated with risk of dementia (AD[b], VaD[b], any dementia) in older Swedish men	Longitudinal (Cognitive and ABPM[a] follow-up after 4 years)	997 (0%)	71 at first exam, 77.6 at second	Swedish men	No dementia at baseline	DSM[b]-IV (dementia); NINCDS-ADRDA[b] (AD[b]); ADDTC[b] (VaD[b])	Not in criteria
Tanaka R (2018) [29]	To assess the relationship between abnormal nocturnal blood pressure profiles and dementia in Parkinson's disease	Cross-sectional	137 (54.0%)	64.1±10.5	Not specified	Not in criteria	Movement Disorder Society Task Force criteria for PDD[b], MMSE[b], HDS-R[b]	Not in criteria
White WB (2018) [30]	Evaluate relationships of clinic, ambulatory, and home BP measurements with WMH[b] burden and mobility/cognitive outcomes in older persons with hypertension	Cross-sectional	199 (54.3%)	81.2±4.1	87.4% Caucasian, 6.5% Black, 4.5% Hispanic/Latino, 1.5% Asian	No dementia	MMSE[b], TMT A&B, Stroop Color and Word Test, Simple Reaction Time, MRI[b]	24h mean systolic hypertension
Xing Y (2021) [12]	To investigate the relationship between ABPM[b] and cognitive impairment in elderly patients and explore the effect on mortality	Cross-sectional	305 (31%)	80.6±7.6	Not specified	Not in criteria	MMSE[b]<27=MCI[b]	Not in criteria
Yamamoto Y (2002) [34]	How ABPM[b] values and MRI[b] findings can predict subsequent development of dementia and vascular events in lacunar infarct patients	Longitudinal (Cognitive follow-up after ~8.9 years)	177 (37.9%)	69.1±8.6	Not specified	Without dementia at baseline	CDR[b], HDSR[b], MRI[b]	Without administration of antihypertensive for >4 weeks
Yamamoto Y (2005) [31]	Investigate relationships between ABPM[b] readings, lacunar infarcts/white matter lesions, and cognitive impairment/VaD[b]	Cross-sectional	200 (39%)	68.8±9.3	Not specified	Without strategic dementia	CDR[b] and HDSR[b]	Without administration of antihypertensive for 2-4 weeks
Yamamoto Y (2011) [32]	Elucidate associations between ABPM[b], cerebral small vessel disease, CKD[b] and cognitive impairment in patients with lacunar infarcts	Cross-sectional	224 (40.2%)	69.8[c]	Not specified	Not in criteria	MMSE[b] ≤24=CI[b], MMSE[b] of 25-27=MCI[b], MRI[b]	Without administration of antihypertensive for >2 weeks
Yaneva-Sirakova T (2016) [33]	Investigate correlation between dipping status and mild cognitive impairment in hypertensive patients	Cross-sectional	439 (63.6%)	64.65±10.15	Not specified	Not in criteria	MoCA[b], MMSE[b]	Hypertensive[d]

[a] Tests listed in the "Cognitive Assessment" column represent only those cognitive assessments that were specifically analyzed in relation to ABPM findings in each study. Additional cognitive or neuroimaging tests may have been conducted in the studies but are not included here if their results were not directly examined in association with ABPM.

[b] Abbreviations: ABPM: Ambulatory Blood Pressure Monitoring; AD: Alzheimer's Disease; ADL: Activities of Daily Living; ADDTC: Alzheimer's Disease Diagnostic and Treatment Centers; AVLT: Auditory Verbal Learning Test; BNT: Boston Naming Test; BP: Blood Pressure; CDR: Clinical Dementia Rating; CDR-SB: Clinical Dementia Rating Sum of Boxes; CERAD: Consortium to Establish a Registry for Alzheimer's Disease; CI: Cognitive Impairment; CKD: Chronic Kidney Disease; CT: Computed Tomography; CVLT: California Verbal Learning Test; DSM: Diagnostic and Statistical Manual of Mental Disorders; DSST: Digit Symbol Substitution Test; ERP: Event-Related Potential; HDS-R: Hasegawa Dementia Scale-Revised; I-ADL: Instrumental Activities of Daily Living; MCI: Mild Cognitive Impairment; MCIS: Mild Cognitive Impairment Screen; MMSE: Mini-Mental State Examination; MoCA: Montreal Cognitive Assessment; MRI: Magnetic Resonance Imaging; NINCDS-ADRDA: National Institute of Neurological and Communicative Disorders and Stroke and the Alzheimer's Disease and Related Disorders Association; NINDS-AIREN: National Institute of Neurological Disorders and Stroke and Association Internationale pour la Recherche et l'Enseignement en Neurosciences; NPS: Neuropsychological Test; PDD: Parkinson's Disease Dementia; SCD: Subjective Cognitive Decline; sMMT: Short Mini-Mental Test; SNSB: Seoul Neuropsychological Screening Battery; SVaD: Subcortical Vascular Dementia; svMCI: Subcortical Vascular Mild Cognitive Impairment; TMT: Trail Making Test; VaD: Vascular Dementia; VFT: Verbal Fluency Test; WMH: White Matter Hyperintensities; WMS: Wechsler Memory Scale; 3MS: Modified Mini-Mental State Examination

[c] calculated from the information in the paper

[d] Diagnosis of hypertension at baseline was not based on ABPM.
